# Metabolic control and hypoglycaemia in people with type 1 diabetes: insulin pump therapy vs. intensified insulin therapy in an unselected cohort in routine care

**DOI:** 10.1186/s13098-021-00700-0

**Published:** 2021-07-23

**Authors:** Guido Kramer, Christof Kloos, Ulrich A. Müller, Gunter Wolf, Nadine Kuniss

**Affiliations:** 1 Department of Internal Medicine III, Endocrinology and Metabolic Disorders, Jena University Hospital, Am Klinikum 1, 07747 Jena, Germany; 2 Outpatient healthcare centre Dr. med. Kielstein, Erfurt, Germany

**Keywords:** Type 1 diabetes, Intensified insulin therapy, Insulin pump therapy, Continuous subcutaneous insulin infusion

## Abstract

**Aims:**

The aim of this study was to compare individuals with type 1 diabetes with continuous subcutaneous insulin infusion (CSII) and intensified insulin therapy (ICT) in routine care regarding metabolic control and treatment satisfaction.

**Methods:**

Individuals with type 1 diabetes (CSII n = 74; ICT n = 163) were analysed regarding metabolic control, frequency of hypoglycaemia and treatment satisfaction (DTSQs range 0–36).

**Results:**

Individuals with CSII (duration of CSII: 14.1 ± 7.2 years) were younger (51.1 ± 15.8 vs. 56.2 ± 16.2 years, p = 0.023), had longer diabetes duration (28.7 ± 12.4 vs. 24.6 ± 14.3 years, p = 0.033), lower insulin dosage (0.6 ± 0.2 vs. 0.7 ± 0.4 IU/kg, p = 0.004), used more frequently short-acting analogue insulin (90.5% vs. 48.5%, p < 0.001) and flash/continuous glucose monitoring (50.0% vs. 31.9%, p = 0.009) than people with ICT. HbA1c was similar between CSII and ICT (7.1 ± 0.8%/54.4 ± 9.1 mmol/mol vs. 7.2 ± 1.0%/55.7 ± 10.9 mmol/mol, p = 0.353). Individuals with CSII had higher frequency of non-severe hypoglycaemia per week (in people with blood glucose monitoring: 1.9 ± 1.7 vs. 1.2 ± 1.6, p = 0.014; in people with flash/continuous glucose monitoring: 3.3 ± 2.2 vs. 2.1 ± 2.0, p = 0.006). Prevalence of polyneuropathy (18.9% vs. 38.0%, p = 0.004) and systolic blood pressure (138.0 ± 16.4 vs. 143.9 ± 17.1 mmHg, p = 0.014) was lower in CSII. Satisfaction with diabetes treatment (26.7 ± 7.3 vs. 26.0 ± 6.8, p = 0.600) did not differ between CSII and ICT.

**Conclusions:**

CSII and ICT yielded comparable metabolic control and treatment satisfaction but CSII was associated with higher incidence of non-severe hypoglycaemia and lower insulin dosage.

## Introduction

Intensified insulin therapy (ICT) and insulin pump therapy (continuous subcutaneous insulin infusion; CSII) are the most common insulin therapies for people with type 1 diabetes. Conventional insulin therapy (CT) with two daily injections of premixed insulin is used in individuals with type 1 diabetes only in exceptional circumstances.

ICT is characterised with multiple injections of short acting human/analogue insulin as well as long/intermediate acting insulin (basal insulin) once or twice daily (“insulin pen therapy”). In contrast, only short acting human/analogue insulin is used for CSII. An insulin pump is a small, computerised device that continuously delivers small insulin doses for basal requirements (hourly basal rate). In addition, patients deliver “bolus insulin” manually according to the amount of carbohydrates or for correction of high blood glucose levels. An insulin pump consists of a control module, an insulin reservoir and an infusion set that includes a cannula and tubing system. A variety of pump systems from different manufacturers is available on the market.

Which strategy of insulin application suits best for individuals with type 1 diabetes to achieve good glycaemic control without hypoglycaemic events is debated. The most frequently used insulin therapy is the ICT [1]. According to current guidelines, ICT is considered the gold standard for the treatment of people with type 1 diabetes [2, 3]. CSII is only prescribed to match for certain conditions, such as a very low insulin requirement [4]. However, the number of individuals with type 1 diabetes and CSII is rising. In Germany, in the years between 2002 and 2014, the number of adults with CSII increased from 13.5 to 31.5% [1] and in children and adolescents from approximately 1% in 1995 to over 40% in 2014 [5]. Especially in the youngest (< 6 years) the number of CSII-users is high with a total share of 79% in 2014 [5]. Thus, inadvertently and without sound evidence this form of insulin therapy already seems to have become standard in children and adolescents.

In comparison to ICT, CSII is promoted to offer more flexibility for the therapy and allows a more physiologic insulin replacement. This should produce better metabolic control (lower HbA1c and less hypoglycaemia) than ICT. Additionally, CSII offers advanced pump functions (e.g. temporary basal rate, bolus variations) to further individualise and optimise insulin therapy. Metabolic control (HbA1c and hypoglycaemic events) are important indicators for successful diabetes management. However, treatment satisfaction and well-being are also very important parameters, if not more meaningful especially for patients.

Therefore, the aim of this study was to compare individuals with type 1 diabetes with ICT and CSII in routine care regarding metabolic control, treatment satisfaction, well-being and social status.

## Patients and methods

All patients with type 1 diabetes attending the University outpatient department of endocrinology and metabolic diseases of the Jena University hospital in the year 2018 were analysed retrospectively in this study. Patients with newly diagnosed diabetes (diabetes duration ≤ 1 month), CSII duration ≤ 1 month, age < 18 years and pregnant women were excluded.

All procedures followed were in accordance with the ethical standards of the responsible committee on human experimentation (institutional and national) and with the Helsinki Declaration of 1975, as revised in 2008.

Laboratory and clinical data were drawn from the digital patient record EMIL [6] and were collected on the day of the survey of the respective participant.

HbA1c was measured using high-performance liquid chromatography (HPLC, TOSOH) with a normal range of 5.0–6.2%. To compare HbA1c values with other studies, HbA1c was adjusted according to the mean normal value of healthy people (5.05%, 32 mmol/mol) according to the DCC trial [7, 8].

Non-severe hypoglycaemia was defined if typical symptoms (e.g. sweating, weak concentration, feeling shaky) were present but disappeared quickly after carbohydrate intake or a plasma glucose ≤ 3.9 mmol/l without typical symptoms [9]. In addition, the patients´ diary was checked. These diaries were analysed by the physician as a part of the consultation every three months.

Severe hypoglycaemia was defined as a condition with necessity of glucagon injection (administered by third party, e.g. relatives) or intravenous glucose injection (administered by medical professionals) with or without hospitalisation according to the guidelines of the German Diabetes Association [10].

Impaired hypoglycaemia awareness was assessed using the validated Gold method [11]. Participants were asked to answer the question “Do you know when your hypos are commencing?” while scoring on a Lickert scale from 1 (“always”) to 7 (“never”). A Gold score ≥ 4 is considered as “impaired hypoglycaemia awareness”.

Treatment satisfaction was assessed with the “Diabetes Treatment Satisfaction Questionnaire status” questionnaire (DTSQs) [12]. DTSQs score ranges from 0 to 36. Higher scores indicate greater treatment satisfaction. The WHO-5 questionnaire was used to assess current well-being. This questionnaire consists of five questions (each item: 0–5 points, total score: 0–25). Higher scores of Wellbeing-5-Index are associated with higher well-being. A score < 7 points indicates a possible depression.

Social status was assessed by a validated questionnaire [13]. Scores range from 3 to 21 points and includes education, highest professional position and household net income.

### Statistical analysis

Statistical analyses were performed with SPSS 25 (IBM Corporation, Armonk, NY, USA). All continuous data are presented as mean ± standard deviation (SD). Categorical data are described by absolute and relative frequencies. An unpaired t-test was used for continuous variables to compare two groups. Fisher’s exact test was performed for categorical variables. Pearson's and Spearman´s correlation coefficient was calculated for assessing relationship between two variables. Significance was defined at the 0.05 level.

## Results

237 people with type 1 diabetes (74 with CSII and 163 with ICT) were included in this trial. The characteristics of the included participants are shown in Table 1. Duration of insulin pump therapy was 14.1 ± 7.2 years (range 0.4–27.7) in individuals with CSII. Insulin pumps of all manufacturers were used.

**Table 1:** Characteristics of study participants

Parameters	Participants			p-value*
	All (n = 237)	CSII (n = 74)	ICT (n = 163)	
Women n (%)	116 (48.9)	47 (63.5)	69 (42.3)	**0.003**
Age (years)	54.6 ± 16.2	51.1 ± 15.8	56.2 ± 16.2	**0.023**
Duration of diabetes (years)	25.9 ± 13.8	28.7 ± 12.4	24.6 ± 14.3	**0.033**
Duration of pump therapy (years)	–	14.1 ± 7.2	–	n/a
Body weight (kg)	81.1 ± 17.4	79.4 ± 17.6	81.9 ± 17.4	0.319
BMI (kg/m^2^)	27.8 ± 5.2	27.6 ± 4.8	27.9 ± 5.4	0.690
HbA1c, DCCT adjusted				
%	7.2 ± 0.9	7.1 ± 0.8	7.2 ± 1.0	0.353
mmol/mol	55.3 ± 10.3	54.4 ± 9.1	55.7 ± 10.9	0.353
Insulin dosage				
Total (IU/day)	52.6 ± 34.9	44.6 ± 22.5	56.2 ± 38.8	**0.004**
Total (IU/kg)	0.6 ± 0.3	0.6 ± 0.2	0.7 ± 0.4	**0.004**
Basal (IU/day)	23.5 ± 16.9	21.9 ± 13.1	24.1 ± 18.4	0.289
Basal (IU/kg)	0.3 ± 0.1	0.3 ± 0.1	0.3 ± 0.2	0.591
Number of insulin injections per day	–	–	5.1 ± 1.2	n/a
Type of short-acting insulin, n (%)				
Analogue	146 (61.6)	67 (90.5)	79 (48.5)	** < 0.001**
Human	91 (38.4)	7 (9.5)	84 (51.5)	
Type of basal insulin, n (%)				
Analogue	–	–	88 (55.7)	n/a
Human	–	–	70 (44.3) ^a^	
Number of people with, n (%)				
SMBG	148 (62.4)	37 (50.0)	111 (68.1)	**0.009**
CGM/FGM	89 (37.6)	37 (50.0)	52 (31.9)	**0.009**
Impaired hypoglycaemia awareness, n (%)	57 (27.0)	21 (28.4)	36 (22.1)	0.623
Gold score (range 1–7)	2.5 ± 1.7	2.7 ± 1.9	2.5 ± 1.6	0.514
Frequency of non-severe hypoglycaemia per week				
In people with SMBG	1.4 ± 1.7	1.9 ± 1.7	1.2 ± 1.6	**0.014**
In people with CGM/FGM	2.6 ± 2.2	3.3 ± 2.2	2.1 ± 2.0	**0.006**
Severe hypoglycaemia past 12 months				
Frequency	0.03 ± 0.18	0.03 ± 0.16	0.04 ± 0.19	0.701
Number of events (people with an event)	8 (8)	2 (2)	6 (6)	1.000
Ketoacidosis past 12 months				
Frequency	0.01 ± 0.09	0	0.01 ± 0.11	0.341
Number of events (people with an event)	2 (2)	0	2 (2)	1.000
Blood pressure				
Systolic (mmHg)	142.1 ± 17.0	138.0 ± 16.4	143.9 ± 17.1	**0.014**
Diastolic (mmHg)	81.5 ± 11.9	81.2 ± 8.3	81.7 ± 13.2	0.711
Pulse (bpm)	75.0 ± 13.1	75.4 ± 12.0	74.8 ± 13.6	0.741
Polyneuropathy, n (%)	76 (32.1)	14 (18.9)	62 (38.0)	**0.004**
Amputation minor/major, n (%)	2 (0.8)	1 (1.4)	1 (0.6)	0.428
Impaired kidney function (eGFR < 60 ml/min), n (%)	41 (17.4)	11 (14.9)	30 (18.4)	0.580
Albumin (mg/l)	52.5 ± 222.0	23.8 ± 45.6	65.7 ± 265.6	0.055
Nephropathy (albumin/g creatinine > 20 mg/g), n (%)	106 (44.7)	32 (43.2)	74 (45.4)	0.777
Dialysis, n (%)	3 (1.3)	1 (1.4)	2 (1.2)	1.000
Living alone, n (%)	44 (19.0)	11 (14.9)	33 (20.2)	0.479
Smoker, n (%)	41 (17.3)	12 (16.2)	29 (17.8)	0.854
Social status (range 3–21)	12.7 ± 4.0	13.5 ± 4.1	12.3 ± 3.9	0.067
DTSQs score (range 0–36)	26.2 ± 6.9	26.7 ± 7.3	26.0 ± 6.8	0.600
Wellbeing-5-Index (range 0–25)	17.0 ± 3.9	16.7 ± 4.0	17.2 ± 3.9	0.409

*BMI* body mass index, *CGM* continuous glucose monitoring, *CSII* continuous subcutaneous insulin infusion, *DTSQs* Diabetes Treatment Satisfaction Questionnaire status, *eGFR* estimated glomerular filtration rate, *FGM* flash glucose monitoring, *ICT* intensified insulin therapy, *IU* international units, *SMBG* self-monitoring of blood glucose

* Between CSII and ICT

^a ^Of 158 individuals (n = 5, no basal insulin)

Individuals with CSII were younger (51.1 ± 15.8 vs. 56.2 ± 16.2 years, p = 0.023), more often female (63.5% vs. 42.3%, p = 0.003) and had longer diabetes duration (28.7 ± 12.4 vs. 24.6 ± 14.3 years, p = 0.033). In addition, study participants with CSII had a lower dose of total insulin (0.6 ± 0.2 vs. 0.7 ± 0.4 IU/kg, p = 0.004) whereas the dose of basal insulin was comparable between CSII and ICT (0.3 ± 0.1 vs. 0.3 ± 0.2 IU/kg, p = 0.591). People with CSII used more frequently short-acting analogue insulin (90.5% vs. 48.5%, p < 0.001) as well as flash/continuous glucose monitoring (50.0% vs. 31.9%, p = 0.009) than people with ICT.

HbA1c was similar between CSII (7.1 ± 0.8%, 54.4 ± 9.1 mmol/mol) and ICT (7.2 ± 1.0%, 55.7 ± 10.9 mmol/mol, p = 0.353). In addition, HbA1c did not differ between users of human short-acting insulin and analogue short-acting insulin in CSII (human 7.0 ± 0.2% vs. analogue 7.1 ± 0.9%, p = 0.510) as well as ICT (human 7.2 ± 0.9% vs. analogue 7.3 ± 1.1%, p = 0.443).

Individuals with CSII had a higher frequency of non-severe hypoglycaemia per week than people with ICT (blood glucose: 1.9 ± 1.7 vs. 1.2 ± 1.6, CGM/FGM: 3.3 ± 2.2 vs. 2.1 ± 2.0, p < 0.05). The number of people with hypoglycaemia unawareness (28.4% vs. 22.1%, p = 0.623) as well as the frequency of severe hypoglycaemia per year (0.03 ± 0.16 vs. 0.04 ± 0.19, p = 0.701) and ketoacidosis per year (0 vs. 0.01 ± 0.11, p = 1.000) differed not between CSII and ICT.

Well-being (16.7 ± 4.0 vs. 17.2 ± 3.9, p = 0.409) as well as satisfaction with diabetes treatment (26.7 ± 7.3 vs. 26.0 ± 6.8, p = 0.600) did not differ between CSII and ICT.

The cohort comprised only two people with amputations (CSII n = 1/74 vs. ICT n = 1/163, p = 0.428) and three people with dialysis (CSII = 1/74 vs. ICT n = 2/163, p = 1.000). Prevalence of impaired kidney function (eGFR < 60 ml/min; 14.9% vs. 18.4%, p = 0.580) as well as nephropathy (albumin/g creatinine > 20 mg/g; 43.2% vs. 45.4%, p = 0.777) was comparable between CSII and ICT. However, prevalence of polyneuropathy was lower in CSII than in ICT (18.9% vs. 38.0%, p = 0.004). After adjustment for age, gender, diabetes duration, social status and HbA1c, ICT was associated with a 2.5 fold higher risk of polyneuropathy than individuals with CSII (p = 0.05). Systolic blood pressure (138.0 ± 16.4 vs. 143.9 ± 17.1 mmHg, p = 0.014) was lower in CSII than in ICT.

## Discussion

The aim of this study was to compare individuals with type 1 diabetes using either an insulin pump (CSII) or a basal bolus regime with insulin pens (ICT) in routine care. CSII and ICT are associated with comparable metabolic control and treatment satisfaction but CSII was associated with lower insulin dosage and higher incidence of non-severe hypoglycaemia.

CSII is commonly regarded as a treatment alternative for people with absolute insulin deficiency (type 1 diabetes) [1]. However, it is more costly compared to therapy with pens and needs skills to be trained for prior to CSII use. In 2014, every third adult with type 1 diabetes used CSII in Germany. One advantage of CSII are advanced functions and features of the insulin pumps like retarded boluses and temporary reduction of the basal rate which possibly could result in fewer hypoglycaemic events (severe and non-severe) and better metabolic control. However, participants of another study conducted with the same cohort of people as this study revealed that these technical aids were scarcely used, although all patients receive a technical instruction at start of pump therapy and participate in a training program. Only 26.4% used the opportunity of temporary basal rate reduction or increase, 11.3% different bolus variations and 2.8% multiple basal rate profiles at least 3 times a week. Furthermore, only 34.7% correctly stated their pump’s range of advanced functions and only 33.3% were able to handle all features [14]. This is in line with the results of a small investigation in Europe indicating that less than half of the patients made use of their additional pump programmes and integrated bolus calculator [15].

HbA1c was comparable between CSII and ICT (statistically not significant difference − 0.1% in favour of CSII). The effect of CSII on metabolic control was assessed in adults in several meta-analysis and a Cochrane review showing a minor advantage for CSII with 0.2 to 0.4% in favour of CSII [16–19]. These meta-analyses also showed the frequency of non-severe hypoglycaemia to be comparable between CSII and ICT. In contrast to these studies, participants with CSII of our study had a higher frequency of non-severe hypoglycaemia than people with ICT. Severe hypoglycaemic events occurred only in 8 occasions numerically but not statistically significant more often in the ICT group.

Metabolic control was similar between CSII and ICT in spite of patients with CSII used more frequently analogue insulin. Furthermore, there was no difference between people with human and analogue short-acting insulin in CSII as well as ICT. This is in line with the results of a Cochrane review by Fullerton showing only a minor (but clinically irrelevant) benefit of short-acting insulin analogues on HbA1c in people with type 1 diabetes (i.e. − 0.15% in favour of insulin analogues) [20]. The partitioning of short-acting and basal insulin was approximately 50:50 for both, CSII and ICT. However, individuals receiving CSII injected overall 12 IU and 0.1 IU/kg per day less than patients treated by ICT whereas the dosage of the basal insulin was also lower in the CSII group which was not statistically significant in this relatively small cohort. People with ICT used more short-acting insulin. However, our study did not investigate what the short-acting insulin was used for: for intake of carbohydrates or correction of high blood glucose levels. This finding is also present in other studies [16, 19]. The Cochrane review by Misso et al. also reported a mean difference of the total daily insulin dose of − 0.1 IU/kg in favour of CSII compared to ICT [19].

Polyneuropathy was more common in people with ICT than CSII. This indicates a positive selection of patients with CSII. Patients with CSII may be more responsible and conscientiously with their diabetes therapy. Maybe CSII-patients have lower blood glucose targets, so they have a higher frequency of hypoglycemic events. In addition, preponderously women used CSII in our cohort. This is consistent with a large population-based study by van den Boom et al. showing the same result in adult individuals in Germany [21]. Women very likely are more diligently overall concerning health issues and thus also with diabetes and diabetes therapy.

Number of individuals with polyneuropathy (but not nephropathy!) was lower in patients with CSII than ICT though duration of diabetes was longer. One reason could be the lower systolic blood pressure in our study participants with CSII. Rosenlund et al. found a significant (but clinically irrelevant) reduction of the albumin-creatinine ratio in CSII patients with no change in mean arterial pressure in both groups. In study participants with CSII, the albumin-creatinine ratio was reduced by 3 mg/g more after four years compared to individuals with ICT [22]. Furthermore, the study by Rosenlund et al. showed that a larger number of patients with microalbuminuria and ICT were fast decliners of eGFR in comparison to CSII. This can be more advantageous regarding outcomes in diabetes. However, more evidence is needed in relation to long-term benefits of CSII (e.g. complications). An observational study in people with type 1 diabetes did not find a difference between CSII and ICT regarding microalbuminuria for almost nine years [23]. The study by Marchand et al. also showed no change in albuminuria four years after switching from CSII and ICT [24]. However, whether diabetes-related complications can be reduced or even prevented by new diabetes technologies cannot be answered so far. Randomised controlled studies are missing.

In summary, no clear advantage exists in favour of one or the other way to apply insulin in type 1 diabetes. Nevertheless, there are some obvious medical reasons to use CSII, like very low insulin requirement or a pronounced dawn-phenomenon. Mostly, such clear-cut reasons in favour of CSII do not exist. Insulin pump therapy is demanding and not suitable for everybody. Additionally it is four times more expensive than ICT in Germany. Insulin therapy needs to fit to each individuals lifestyle and circumstances of living and it must be checked individually whether the conditions and indications for insulin pump therapy are met as well as if the individual benefits from such a therapy. Therefore, patients should be actively involved in decision making according to the patients’ lifestyle and preferences and carefully guided through the initiation phase. Involving patients to find the optimal insulin strategy may lead to better quality of life and adherence of their therapy. Successful diabetes management is often measured by metabolic control. However, treatment satisfaction and well-being are also very important parameters, especially for patients. It decides whether patients choose one or the other therapy option. Treatment satisfaction and well-being were comparable between ICT and CSII in our study. This suggests that ICT and CSII are preferred by different patient groups and both are important in diabetes therapy. Social status does not appear to be a reason to choose pen or pump therapy, because the score was comparable between both study groups.

A strength of our study is the well-characterised cohort of people type 1 diabetes. All used questionnaires are evaluated and validated. In comparison to randomised controlled trails, this study showed real-world data of routine care. However, our study has some limitations, e.g. the limited number of study participants. This study is not a longitudinal investigation whereby the course of therapy cannot be compared between CSII and ICT. In addition, due to the retrospective study design, no causal relationships can be conceived. Furthermore, retrospective studies could bias the analysis and may introduce selection bias or information bias. For example, the higher prevalence of patients with polyneuropathy in the ICT group indicates a positive selection of individuals with CSII.

## Conclusions

CSII and ICT are comparable therapy strategies in people with type 1 diabetes. Both therapies yielded comparable metabolic control and treatment satisfaction but CSII was associated with higher incidence of non-severe hypoglycaemia and lower insulin dosage. Apart from obvious medical reasons should individuals with type 1 diabetes be actively involved in decision making to find the optimal insulin strategy.

## Data Availability

All data generated or analysed during this study are included in this published article.

## Abbreviations

BMI: Body Mass Index
CSII: Continuous subcutaneous insulin infusion
HbA1c: Glycated haemoglobin
ICT: Intensified insulin therapy
